# Anticipation and violated expectation of pain are influenced by trait rumination: An fMRI study

**DOI:** 10.3758/s13415-018-0644-y

**Published:** 2018-09-24

**Authors:** Gyongyi Kokonyei, Attila Galambos, Andrea Edit Edes, Natalia Kocsel, Edina Szabo, Dorottya Pap, Lajos R. Kozak, Gyorgy Bagdy, Gabriella Juhasz

**Affiliations:** 1 SE-NAP2 Genetic Brain Imaging Migraine Research Group, Hungarian Academy of Sciences, Semmelweis University, Budapest, Hungary; 2 Institute of Psychology, ELTE Eötvös Loránd University, Izabella street 46, Budapest, H-1064 Hungary; 3 Department of Pharmacodynamics, Faculty of Pharmacy, Semmelweis University, Budapest, Hungary; 4 Doctoral School of Psychology, ELTE Eötvös Loránd University, Budapest, Hungary; 5 MTA-SE Neuropsychopharmacology and Neurochemistry Research Group, Hungarian Academy of Sciences, Semmelweis University, Budapest, Hungary; 6 MR Research Center, Semmelweis University, Budapest, Hungary; 7 Neuroscience and Psychiatry Unit, The University of Manchester, Manchester, UK; 8 Manchester Academic Health Sciences Centre, Manchester, UK

**Keywords:** Expectation, Pain anticipation, fMRI, Rumination, Violation, Conditioning

## Abstract

Rumination – as a stable tendency to focus repetitively on feelings related to distress – represents a transdiagnostic risk factor. Theories suggest altered emotional information processing as the key mechanism of rumination. However, studies on the anticipation processes in relation to rumination are scarce, even though expectation in this process is demonstrated to influence the processing of emotional stimuli. In addition, no published study has investigated violated expectation in relation to rumination yet. In the present study we examined the neural correlates of pain anticipation and perception using a fear conditioning paradigm with pain as the unconditioned stimulus in healthy subjects (*N* = 30). Rumination was assessed with the 10-item Ruminative Response Scale (RRS). Widespread brain activation – extending to temporal, parietal, and occipital lobes along with activation in the cingulate cortex, insula, and putamen – showed a positive correlation with rumination, supporting our hypothesis that trait rumination influences anticipatory processes. Interestingly, with violated expectation (when an unexpected, non-painful stimulus follows a pain cue compared to when an expected, painful stimulus follows the same pain cue) a negative association between rumination and activation was found in the posterior cingulate cortex, which is responsible for change detection in the environment and subsequent behavioral modification. Our results suggest that rumination is associated with increased neural response to pain perception and pain anticipation, and may deteriorate the identification of an unexpected omission of aversive stimuli. Therefore, targeting rumination in cognitive behavioral therapy of chronic pain could have a beneficial effect.

**Electronic supplementary material:**

The online version of this article (10.3758/s13415-018-0644-y) contains supplementary material, which is available to authorized users.

Rumination, representing recurrent and repetitive distress-related thoughts (Nolen-Hoeksema, Wisco, & Lyubomirsky, 2008; Watkins, 2008) has been considered as a transdiagnostic risk factor in the development and maintenance of several psychopathological disorders including mood, anxiety, eating, and addiction disorders (Nolen-Hoeksema et al., 2008). However, ruminative thoughts are not restricted to mental health disorders, but can also be present in everyday cognitions (Ottaviani, Medea, Lonigro, Tarvainen, & Couyoumdjian, 2015) and are associated with elevated psychopathological symptoms (Aldao, Nolen-Hoeksema, & Schweizer, 2010) and lower well-being in healthy people. In addition, the role of perseverative cognitions – such as rumination – in shaping somatic and psychological health in chronic somatic conditions has been proposed (Brosschot, Gerin, & Thayer, 2006; Soo, Burney, & Basten, 2009), and our previous study in migraine headache also supported this notion (Kokonyei et al., 2016). Studies in chronic pain, for instance, also suggest that rumination on negative emotions and pain occurs frequently (Edwards, Tang, Wright, Salkovskis, & Timberlake, 2011) and explains individual differences in mental health and functioning (McCracken, Barker, & Chilcot, 2014).

To understand the contribution of rumination to mental and somatic health, the identification of mechanisms/pathways by which rumination exerts its effects is required. In a recent theory, Koster and colleagues (Koster, De Lissnyder, Derakshan, & De Raedt, 2011) have proposed altered information processing as the key underlying mechanism of rumination. Indeed, several studies – using among others eye-tracking (e.g., Duque, Sanchez, & Vazquez, 2014; Owens & Gibb, 2017), and reaction-time paradigms (e.g., Grafton, Southworth, Watkins, & MacLeod, 2016), and neuroimaging methodology (e.g., Vanderhasselt, Kuehn, & De Raedt, 2011; Vanderhasselt et al., 2013) showed that biased emotional and attentional processing of negative (aversive) information is associated with rumination. Attentional control deficits in rumination seem to be mood-independent (for a review, see Koster et al., 2011), pointing to a possible mechanism through which rumination exerts its effect even in healthy people.

Despite the extant knowledge on the relation between rumination and emotional processing and attentional control of these processes, several gaps exist. Expectations associated with negative and positive stimuli (events) may influence processing of emotional information and outcomes. However, the anticipation phase in emotional processing in relation to rumination has been studied rarely, and mainly in association with depression (Schiller, Minkel, Smoski, & Dichter, 2013; Whitmer & Banich, 2012). On the other hand, one recent fMRI study (Kocsel et al., 2017) demonstrated that trait rumination was associated with increased activation in brain areas related to the Salience Network to reward cues compared to loss cues in a variant of classic monetary incentive delay (MID) task (Dillon et al., 2008; Pizzagalli et al., 2009).

The anticipation process can be studied in conditioning paradigms where cues predict positive or negative outcomes with some probabilities. Learned association between cues and outcomes transforms sensory characteristics of a cue into emotional meaning on a neural level (Lobanov, Zeidan, McHaffie, Kraft, & Coghill, 2014), which then directs subsequent perception of stimuli. For example, applying a mediation analysis, a study using painful heat stimuli demonstrated that brain activity associated with pain predictive cues had an effect on perceived pain both on a subjective and on a neural level (Atlas, Bolger, Lindquist, & Wager, 2010).

Pain as an aversive – unconditioned – stimulus is frequently used in a conditioning paradigm, since it elicits fear. Cues predicting pain are reliably associated with widespread brain activation (see Palermo, Benedetti, Costa, & Amanzio, 2015), and the anticipatory phase is usually perceived as an unpleasant – dreadful – period (Berns et al., 2006). Pain predictive cues are appraised as threats, thus they catch the individual’s attention (Brown, Seymour, Boyle, El-Deredy, & Jones, 2008), leading to preparatory responses and/or avoidance behavior. This process could work only if we learn the association between cues and outcomes, and store this knowledge in our memory. It is important to note that cue-based information processing can be influenced by several factors and may be altered in different psychopathologies (Baas, 2013; Stice, Spoor, Bohon, Veldhuizen, & Small, 2008).

In addition, conditioning paradigms offer the possibility to investigate neural activations to the detection of mismatch between the expected and experienced emotional stimulus (D'Astolfo & Rief, 2017). From a clinical point of view, detection of discrepancy between expectations and real experience is essential for over-riding existing beliefs that exert their effects in a top-down manner. Based on clinical observations and treatment studies, reducing rumination is difficult (see Watkins, 2016): even after a significant improvement in mood due to therapeutic intervention, rumination remains at a high level (Riso et al., 2003; Roberts, Gilboa, & Gotlib, 1998), suggesting that detection of violations of negative expectations may be impaired in persons with high ruminative tendencies. Our notion is indirectly supported by other types of evidence: cognitive studies, for instance, show that switching attention from negative emotional information to non-emotional information in working memory is impaired in rumination (Koster, De Lissnyder, & De Raedt, 2013).

Based on the above-mentioned results, we suggest that not only does rumination affect the perception of emotional stimuli, but the anticipation phase of emotional processing is also influenced by individual differences in rumination. In our study we used a fear conditioning paradigm with pain as the unconditioned stimulus, and our aim was to understand the relationship between rumination and neural processing of conditioned pain in healthy subjects when pathological mood and chronic pain do not interfere with brain activation patterns. Thus, in the present study, we examined the neural correlates of pain anticipation and perception.

Based on previous evidence on altered emotional processing (Koster et al., 2011), we hypothesized that inter-individual differences in rumination will be associated with perception of pain on a neural level, since pain is an aversive and highly salient stimulus. We expected exaggerated neural response to painful stimuli compared to non-painful ones in the pain-processing network (PPN), including the somatosensory areas, insula, anterior cingulate cortex (ACC), and thalamus (Apkarian, Bushnell, Treede, & Zubieta, 2005; Tracey & Mantyh, 2007), among participants who score higher on a trait rumination measure. However, it is unknown whether this excessive response to aversive stimuli in relation to rumination exists in the anticipation phase of emotional processing. Cues were presented for a longer period (mean duration was 9 s), which allowed conscious cognitive expectations to activate and work in the anticipation period. It is worth mentioning that based on Bubic and colleagues’ paper (Bubic, von Cramon, & Schubotz, 2010) we used the term “anticipation” when referring to the process itself; namely the processing of a cue that has been associated with an outcome. Expectation refers to the representation of the cue-outcome association. We hypothesized that pain anticipation would relate to rumination, and expected that activation of brain areas involved both in detection of salience and in pain anticipation/processing – particularly the anterior insula and ACC (see Menon, 2015) – would correlate with trait rumination positively.

We also suggest that rumination deteriorates, or at least influences, the detection of discrepancy between expectations and experience. Therefore, a partial-reinforcement schedule was chosen that allowed us to compare neural responses to discrepancy between cues and stimuli (see Fig. 1). Particularly, we hypothesized that contrasting the omission of painful stimuli and delivered painful stimuli – as an example for violated expectation – would be associated with rumination.

**Fig. 1:**
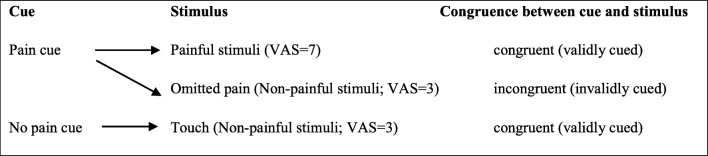
Experimental design. *VAS* Visual Analog Scale. Fifteen trials for pain cue (ten for congruent and five for incongruent) and 15 trials for no pain cue

## Methods

### Participants

Thirty-eight healthy, right-handed subjects aged 18–38 years (23 females and 15 males, mean age ± *SD*: 25.79 ± 4.17) recruited via newspapers and university advertisements participated in the present study. All of the subjects had normal or corrected-to-normal vision. Exclusion criteria were any current or past serious medical, neurological, or Axis I psychiatric disorders and psychotropic medication use. All participants were first screened by a trained researcher using the Mini International Neuropsychiatric Interview (Sheehan et al., 1998) and then participated in a medical examination by experienced neurologist and psychiatrist researchers. The study was approved by Scientific and Research Ethics Committee of the Medical Research Council (Hungary) and written informed consent was received from all subjects in accordance with the Declaration of Helsinki.

Eight subjects were excluded from the study: five by reason of technical problems, one due to excessive movement in the scanner, and two because they did not understand the correspondence between visual signals and electric stimuli according to the post-session interview (see below). Altogether, 30 participants (18 women, mean age ± *SD*: 25.97 ± 4.04 years) were included in the analysis.

### Self-report measures

Rumination was measured by a 10-item Ruminative Response Scale (RRS-10, Treynor, Gonzalez, & Nolen-Hoeksema, 2003), capturing trait-like ruminative thoughts when experiencing low mood. Items are answered on a 4-point Likert-type scale (1 = *almost never*, 2 = *sometimes*, 3 = *often*, 4 = *almost always*). We followed the recommendation of Whitmer and Gotlib (2011), and used the sum score of the scale.

Participants also completed the trait anxiety version of State-Trait Anxiety Inventory (STAI; Spielberger, Gorsuch, Lushene, Vagg, & Jacobs, 1983). The scale comprises 20 items scored on a 4-point Likert-type scale (1 = *almost never* to 4 = *almost always*). STAI-T – along with RRS-10 – was completed some days before the measurement.

Current depressive symptoms – covering the past several days – were assessed by the 20-item Zung Self-Rating Depression Scale (Zung, 1965). Items are answered on a 4-point Likert-type scale (from 1 = *a little of the time* to 4 = *most of the time*). ZSDS was filled out on the scan day.

Internal consistency (Cronbach-α) of all self-report measures was excellent (RRS-10 = 0.80, ZSDS = 0.83, STAI-T = 0.91).

### Psychological task

Two electric stimuli were applied to the dorsum of the right hand. The electrode was connected to Digitimer boxes (Digitimer DS7A, Digitimer Ltd, Welywyn Garden City, UK), one applied the non-painful stimuli, and the other to the painful stimuli. Inputs to Digitimer boxes were controlled by an E-Prime script (Psychology Software Tools, Inc., Pittsburgh, PA, USA) to ensure properly delivered stimuli.

The shocks were calibrated individually before the scanning session using a stair-case method. We used a 10-point visual analog scale (VAS) and participants were asked to rate the stimuli, which were applied to the dorsum of the right hand. When they rated a stimulus as 3 (non-painful stimulus, VAS = 3, currents ranging from 0.1 to 2.8 mA), we set it as a non-painful one. Then we continued the procedure to ascertain the stimulus rated as 7 (as a painful but not an intolerable shock, VAS = 7, currents ranging from 0.35 to 4.2 mA). Only these two stimuli were used during the fMRI session. Electric shocks were pulses of 2-ms duration similar to the study by Spoormaker and colleagues (Spoormaker et al., 2011).

Subjects were told that they would see shapes of two kinds on the screen, a green triangle and a red square, and every shape will be followed by an electric stimulus to the back of the right hand. They were instructed to lie still during the session and pay attention to the screen. The experimental paradigm contained 30 trials in total in which one shape (e.g., green triangle, in 15 trials) was always followed by a non-painful stimulus and the other visual signal (e.g., red square, in 15 trials) was followed by a painful stimulus on all but five trials. In these five trials, a non-painful stimulus (VAS rating = 3) followed the pain cue (we will refer to them as omitted pain trials) (see Fig. 1). The visual signal was always preceded by a white fixation cross presented for 1 s, and was immediately followed by one of the cues. The duration of visual stimuli was pseudorandomized, ranging from 6 to 12 s (average = 9.3 s). A black screen lasting 30 s was shown during the inter-trial interval. Stimulus presentations were delivered by E-Prime 2.0 (Psychology Software Tools, Inc., Pittsburgh, PA, USA).

After the task in a post-session interview participants were asked whether they found any correspondence between cues and subsequent stimuli.

### fMRI acquisition

Functional MRI data acquisition was performed on a 3T MRI scanner (Achieva 3T, Philips Medical Systems, Best, The Netherlands) using a BOLD-sensitive T2*-weighted echo-planar imaging sequence (repetition time [TR] = 2,500 ms, echo time [TE] = 30 ms, field of view [FOV] = 240 × 240 mm^2^) with 3 mm × 3 mm in-plane resolution and contiguous 3-mm slices providing whole-brain coverage. A series of high-resolution anatomical images were also acquired during the first functional imaging session using a T1-weighted 3D TFE sequence with 1 × 1 × 1 mm resolution.

### Statistical analysis of self-report data

Demographic and self-report data were analyzed in SPSS version 23.0 (IBM SPSS, IBM Corp, Armonk, NY, USA) using t-tests and correlation analyses and a two-tailed p<0.05 threshold.

### fMRI data analysis

#### Preprocessing

Statistical Parametrical Mapping (SPM12) analysis software package (Wellcome Department of Imaging Neuroscience, Institute of Neurology, London, UK; http://www.fil.ion.ucl.ac.uk/spm12/) implemented in Matlab 2015b (Math Works, Natick, MA, USA) was used to analyse all imaging data. Functional images were pre-processed, which included realignment, co-registration to the structural image, segmentation, normalization in Montreal Neurological Institute (MNI) space, and spatial smoothing with an 8-mm full width half-maximum Gaussian kernel.

#### First level model

For the first level analysis, BOLD (blood oxygenation level-dependent) hemodynamic responses were modelled in a general linear model. In the event-related single subject analysis: fixation cross, each of the two cues (pain cue and no-pain cue) and the three outcomes (touch, pain, omitted pain; see Fig. 1) were modelled as separate regressors of interest. High-pass temporal filtering with a cut-off of 128 s was included in the model to remove the effects of low-frequency physiological noise, and serial correlations in data series were estimated using an autoregressive AR (1) model.

The motion outliers (threshold of global signal > 3 *SD* and motion > 1 mm) identified with the Artifact Detection Tools (ART www.nitrc.org/projects/artifact_detect/) and the six motion parameters were used as regressors of no interest in the fMRI model. We had to exclude one participant due to excessive movement revealed by the ART (number of outliers > 15%). In the last step of pre-processing, quality of images was visually inspected.

**Fig. 2:**
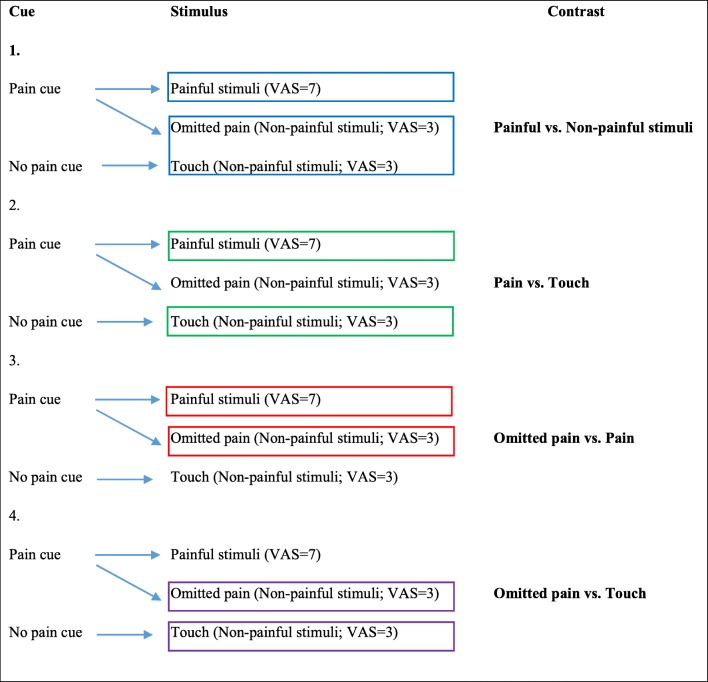
Contrasts for pain period activation. The last two modelled the violated expectations

In our classical conditioning design, stimuli occurred immediately after the offset of cues, therefore we used only those contrasts in which collinearity (correlations) between regressors did not cause a problem (r^2^ < 0.03). Thus, first-level analysis was performed on each participant focusing on the significant BOLD signal responses to anticipation of pain (pain cue vs. no pain cue) and to perception of pain. We used four contrasts for the pain perception period, the last two modelling the violated expectations (see Fig. 2).

1. We analyzed neural response to pain versus no pain, regardless of cues [(painful vs. non-painful stimuli, reflecting painful stimuli (VAS = 7) vs. all non-painful stimuli (all VAS = 3 stimuli: omitted pain + touch)].
2. We also analyzed brain response to pain, using contrast with cue-congruent stimuli (pain vs. touch).
3. Violated expectation was modelled by omitted pain versus pain contrast, characterized by the same expectations but with different intensities.
4. Violated expectation was also modelled with omitted pain versus touch contrast, characterized by different expectations, but with the same intensity.

#### Second level analysis

Every contrast served as a dependent factor and the individual rumination scores were included in the analysis as a covariate. Whole-brain analyses were carried out at a p<0.001 uncorrected level and cluster-level family-wise error-corrected pFWE <0.05 values were reported as significant (with a cluster size >10). Activated clusters were identified anatomically using the automated anatomical labelling atlas (aal; Tzourio-Mazoyer et al., 2002). Statistical maps were visualized on the MNI 152 template brain provided in MRIcroGL (http://www.mccauslandcenter.sc.edu/mricrogl/).

Additional analyses: In our study, correlation between rumination and current depressive mood was unrelated (see below), but we repeated our analyses including depression as an additional covariate to check whether results remain the same (results are shown in the Online Supplementary Materials).

Previous studies on anticipation of pain have demonstrated that trait anxiety has an effect on this phase (Grupe & Nitschke, 2013), therefore we also checked whether results changed if trait anxiety was added as a covariate (results are shown in the Online Supplementary Materials).

## Results

### Self-reported and behavioral results

Mean RRS-10 score was 22.03 (*SD* = 5.36), mean ZSDS score was 33.63 (*SD* = 6.12), and mean STAI-T score was 39.27 (*SD* = 9.97). The correlation between rumination and current depressive symptoms, and correlation between rumination and trait anxiety were only marginally significant (*r* = 0.35, *p* < 0.06; *r* = 0.33, *p* < 0.08, respectively). There were no significant sex differences in either rumination or in depression/trait anxiety scores.

### fMRI results

#### Task-related activations

Details of task-related activations are shown in the Online Supplementary Material.

##### Activation changes during pain anticipation

The pain anticipation contrast (pain cue – no pain cue) showed increased activation only in calcarine and lingual gyrus and decreased activation in two clusters of occipital cortex bilaterally (see Supplementary Table S1 and Fig. S1).

##### Activation changes to painful stimuli

Contrasting painful versus non-painful and contrasting painful with validly cued non-painful stimuli (pain vs. touch) led to widespread activation in brain areas involved in pain perception including the insula, thalamus, inferior frontal gyrus, middle cingulate gyrus, and in the midbrain at the level of periaqueductal gray (see Supplementary Table S2 and Fig. S2). As expected, when pain versus omitted pain was analyzed a largely overlapping activation was found with pain versus touch activation (see Supplementary Fig. S3).

##### Violated expectation

Violated expectation of pain (omitted pain) in comparison to pain was associated with increased BOLD signal in one cluster in parietal cortex (see Supplementary Table S3).

When we compared the omitted pain versus touch stimulation we found deactivation in the right calcarine and lingual gyrus (see Supplementary Table S3). It is worth noting that this deactivation partially overlapped with the activation to pain cue versus no pain cue (see Supplementary Fig. S1).

#### Regression analyses with rumination scores

##### Anticipation

**Fig. 3:**
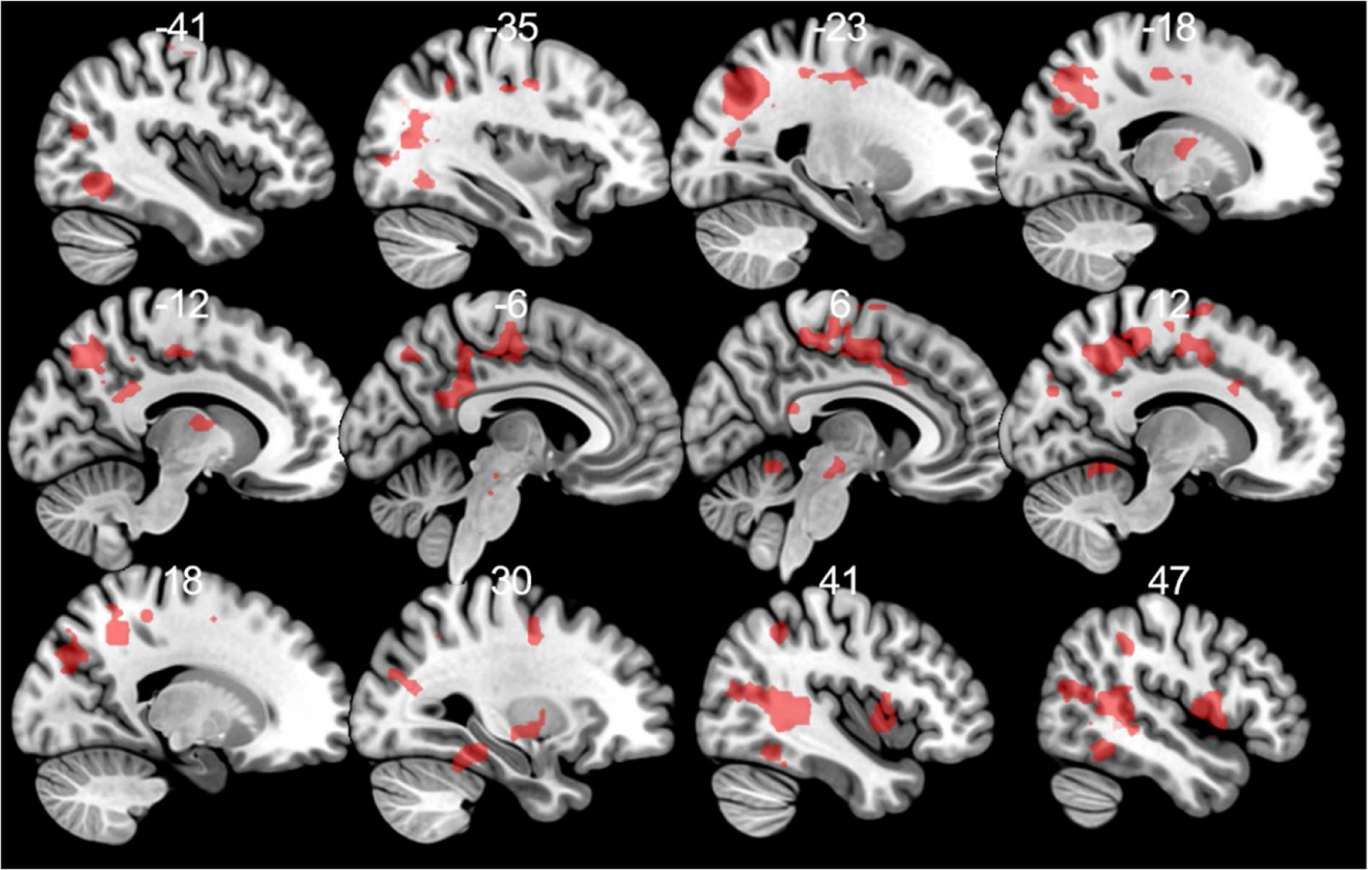
Anticipation of pain in relation to trait rumination at a *p* < 0.001 threshold for illustrative purposes

Rumination was positively correlated with BOLD activity to pain anticipatory cue compared to no pain cue in seven clusters with peaks in the left inferior and right superior temporal gyrus, in the bilateral inferior and left superior parietal lobules, and in the frontal lobe (paracentral lobule, middle frontal gyrus, SMA) extending to the midcingulate and in the left posterior cingulum extending to the midcingulate and in the right insula/Rolandic Operculum and putamen (see Table 1 and Fig. 3).

**Table 1:** Anticipation of pain in relation to trait rumination

Contrasts	RRS	Cluster size (voxels)	Region	Side	Peak T- value	MNI coordinates		
						x	y	z
Pain cue - No pain cue	+	91	Inferior temporal gyrus	L	6.72	-42	-61	-7
			Inferior temporal gyrus	L	4.90	-48	-61	-13
		197	Insula	R	6.00	45	5	-1
			Putamen	R	4.63	33	-10	-4
			Rolandic operculum	R	4.56	54	-10	14
			Rolandic operculum	R	4.53	48	5	11
			Temporal pole	R	3.54	57	5	-13
			Putamen	R	3.49	30	-1	5
		610	Paracentral lobule	R	5.55	12	-31	56
			Middle frontal gyrus	L	4.95	-27	-10	47
			Na	L	4.77	-18	-22	47
			Supplementary motor area	R	4.72	3	-1	53
			Postcentral gyrus	L	4.60	-27	-34	50
			Precuneus	R	4.60	15	-55	44
			Supplementary motor area		4.54	0	-16	53
			Middle cingulate cortex	L	4.35	-9	-22	47
			Precuneus	R	4.30	15	-49	41
			Middle cingulate cortex	R	4.30	12	17	29
			Frontal superior gyrus	R	4.20	15	-1	53
			Paracentral lobule	L	4.11	-3	-16	65
			Middle cingulate cortex	R	4.09	9	-10	47
			Superior parietal lobule	R	3.87	18	-52	59
			Middle cingulate cortex	R	3.85	6	11	38
			Supplementary motor area	R	3.82	6	-22	65
		596	Superior temporal gyrus	R	5.50	42	-40	5
			Superior temporal gyrus	R	5.43	30	-67	17
			Middle temporal gyrus	R	5.29	45	-61	14
			Superior temporal gyrus	R	5.25	57	-37	8
			Superior temporal gyrus	R	5.07	21	-70	32
			Fusiform gyrus	R	5.00	30	-40	-19
			Inferior temporal gyrus	R	4.98	45	-52	-16
			Superior temporal gyrus	R	4.65	42	-49	14
			Middle occipital gyrus	R	4.38	33	-76	23
			Middle temporal gyrus	R	4.04	48	-73	17
			Middle temporal gyrus	R	3.92	42	-70	17
			Superior temporal gyrus	R	3.84	63	-28	5
			Cuneus	R	3.77	9	-76	26
		474	Superior occipital lobule	L	5.18	-21	-70	29
			Superior parietal lobule	L	4.88	-15	-70	47
			Superior parietal lobule	L	4.67	-24	-73	14
			Middle occipital gyrus	L	4.62	-36	-67	23
			Inferior parietal lobule	L	4.60	-24	-67	41
			Middle occipital gyrus	L	4.60	-36	-70	17
			Precuneus	L	4.49	-12	-61	47
			Precuneus	L	4.41	-15	-58	50
			Middle occipital gyrus	L	4.35	-36	-82	5
			Middle occipital gyrus	L	4.34	-30	-73	38
			Precuneus	L	4.32	-18	-58	35
			Inferior parietal lobule	L	4.14	-30	-52	38
			NA	L	4.13	-33	-55	26
			NA	L	3.61	-33	-58	14
			Middle occipital gyrus	L	3.60	-45	-70	23
		85	Inferior parietal lobule	R	4.25	51	-40	41
			Inferior parietal lobule	R	3.83	39	-49	47
		100	Posterior cingulate cortex	L	4.50	-6	-49	23
			Posterior cingulate cortex	L	4.35	-6	-40	29
			NA	L	4.34	-12	-43	29
			Middle cingulate cortex	L	3.96	-6	-43	47
			Middle cingulate cortex	L	3.78	-3	-43	38

Analyses are conducted using *p* < 0.001 primary and *p*(FWE) = 0.05 secondary cluster extent threshold. *RRS* 10-item Ruminative Response Scale, + positive correlation, *R* right, *L* left, *NA* coordinates are not in AAL

We repeated our analysis with possible confounding variables. First, when current depressive symptoms were controlled for, BOLD activity in six clusters with peaks in insula, temporal, parietal, and frontal lobes (paracentral lobule, postcentral gyrus, SMA) remained significant; however, the size of these clusters decreased compared to our analysis when current depressive symptoms were not controlled for. In addition, significant activation to pain cue in the posterior cingulate cortex (PCC) disappeared (see Supplementary Table S4). Second, we also repeated the analysis adding trait anxiety as a covariate. It did not alter our results to a great extent; however, the significant BOLD increase in the insula in relation to rumination disappeared, but additional activation in the inferior frontal gyrus was found (see Supplement Table S5).

##### Intensity of stimulus (regardless of cues)

**Fig. 4:**
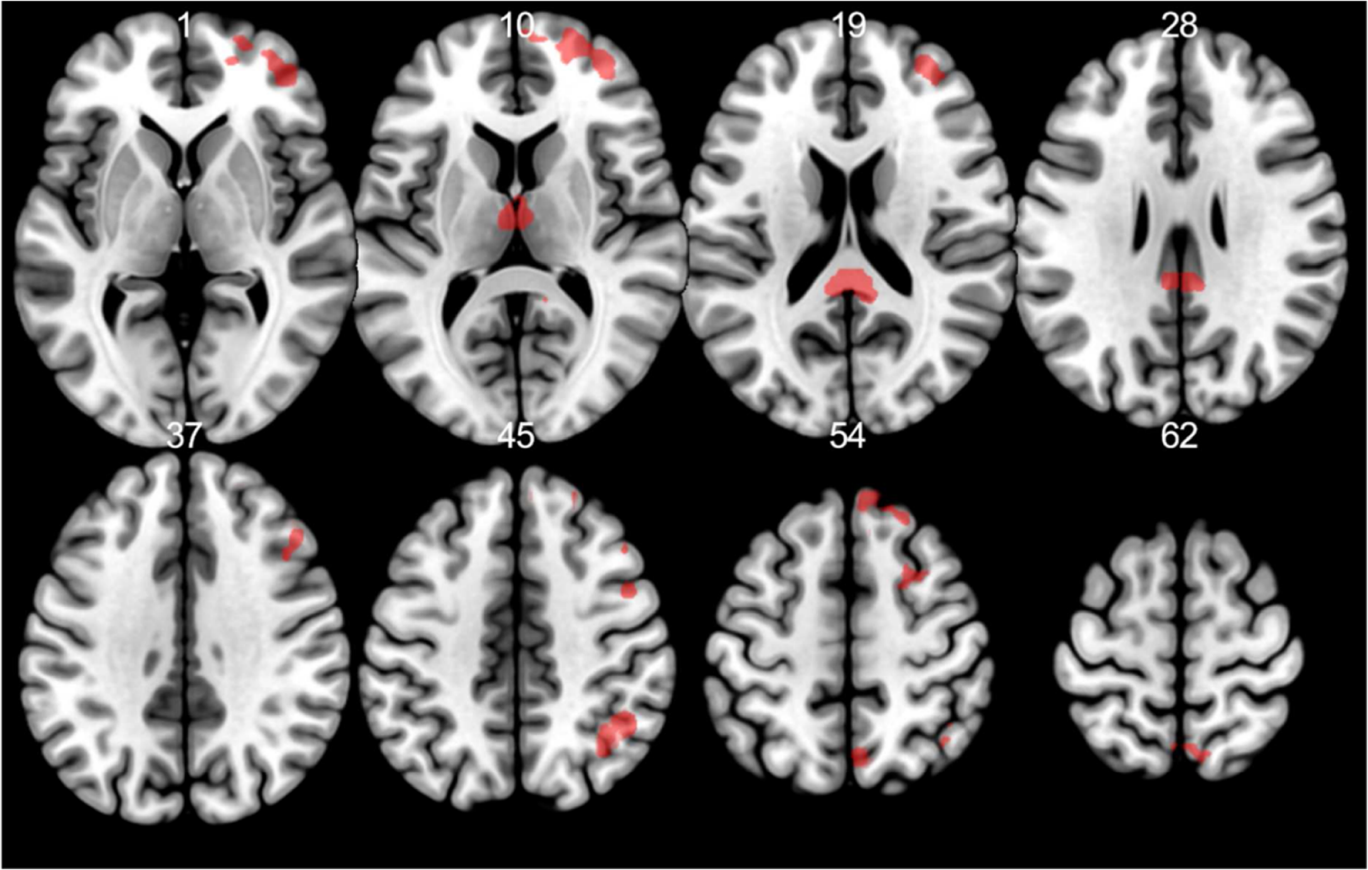
Painful vs. non-painful stimuli in relation to trait rumination at *p* < 0.001 threshold for illustrative purposes with a minimum cluster size of 10 voxels

Regardless of cues, activation to painful stimuli (VAS = 7) versus non-painful stimuli (VAS = 3) was related to rumination in clusters with peaks in the right frontal and parietal lobules (angular gyrus) and in the bilateral thalamus (see Table 2 and Fig. 4).

**Table 2:** Perception of pain in relation to trait rumination

Contrast	RRS	Cluster size (voxels)	Region	Side	Peak T-value	MNI coordinates		
						x	y	z
Pain - Touch			No significant activation					
Painful - Non-painful stimuli	+	100	NA	R	5.88	3	-37	20
			Precuneus	R	3.94	15	-46	14
		163	Superior frontal gyrus	R	5.33	21	59	8
			Superior frontal gyrus	R	4.81	24	62	2
			Middle frontal gyrus	R	4.55	39	53	2
			Middle frontal gyrus	R	4.38	45	50	2
			Superior frontal gyrus	R	4.36	36	50	20
			Middle frontal gyrus	R	4.34	39	53	20
			Middle frontal gyrus	R	4.05	48	47	5
			Superior frontal gyrus	R	3.91	21	53	-1
		67	Angular gyrus	R	4.67	36	-64	47
			Angular gyrus	R	4.64	42	-58	50
		63	Thalamus	L	4.52	-3	-16	14
			Thalamus	R	4.20	6	-13	14
			Thalamus	L	3.93	-15	-22	17
		65	Superior frontal gyrus	R	4.38	21	35	56
			Superior frontal gyrus	R	4.34	24	44	44
			Superior medial frontal gyrus	R	4.31	6	41	53
			Superior medial frontal gyrus	R	4.29	12	44	53
			Superior frontal gyrus	R	4.23	18	38	53
			NA	R	4.05	6	38	59
			Superior medial frontal gyrus	R	3.97	9	23	56

Analyses are conducted using *p* < 0.001 primary and *p*(FWE) = 0.05 secondary cluster extent threshold. *RRS* 10-item Ruminative Response Scale, + positive correlation, *R* right, *L* left, *NA* coordinates are not in AAL

When controlling for depressive symptoms, we found that the size of the activation in the inferior parietal lobule and middle and superior frontal gyrus increased, and extended to the inferior frontal gyrus, but significant activation of the thalamus disappeared (see Supplementary Table S6).

##### Intensity of the stimulus - congruency between cues and stimuli

Interestingly, when we analyzed validly cued stimuli, no correlation between rumination and BOLD activity was found (see Table 2). However, when current depressive symptoms were controlled for, rumination correlated positively with activation in one cluster with peaks in the middle and superior frontal gyrus (see Supplementary Table S6).

##### Violated expectations – discrepancy between cues and stimuli

**Fig. 5:**
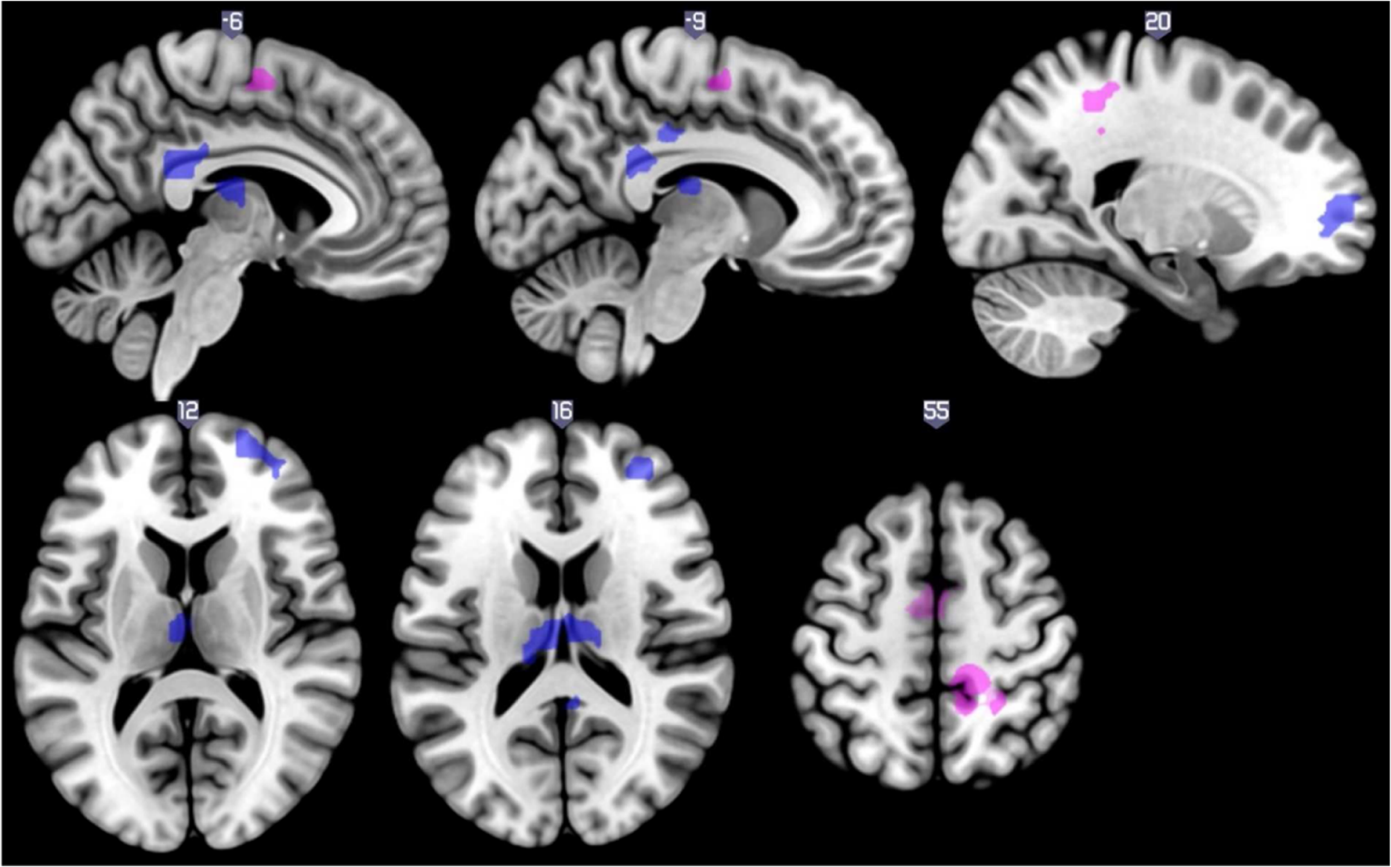
Neural response to discrepancy between cues and stimuli related to rumination negatively at *p* < 0.001 threshold for illustrative purposes with a minimum cluster size of 10 voxels. *Blue*: omitted pain vs. pain, *purple*: omitted pain vs. touch

First, we contrasted omitted pain stimuli versus pain stimuli, and both were preceded with pain cues but they differed in the intensity of electrical stimulation. We found that BOLD signals in the left thalamus, right superior frontal gyrus, and left posterior cingulate (extending to middle cingulate cortex) were negatively correlated with rumination score (see Table 3 and Fig. 5).

**Table 3:** Neural response to discrepancy between cues and stimuli related to rumination

Contrast	RRS	Cluster size (voxels)	Region	Side	Peak T-value	MNI coordinates		
						x	y	z
Omitted pain - Pain	-	86	Thalamus	L	5.68	-3	-16	17
			Thalamus	L	3.99	-12	-25	17
		159	Posterior cingulate cortex	R	5.27	3	-37	23
			Middle cingulate cortex	L	3.99	-9	-31	38
		97	Superior frontal gyrus	R	4.87	21	59	8
			Superior frontal gyrus	R	4.72	24	62	2
			Superior frontal gyrus	R	4.64	18	53	2
			Superior frontal gyrus	R	4.59	27	59	11
			Middle frontal gyrus	R	4.34	36	50	20
			Middle frontal gyrus	R	3.90	36	50	11
Omitted Pain - Touch	-	137	Postcentral gyrus	R	5.27	27	-43	50
			Postcentral gyrus	R	5.20	15	-34	56
			Postcentral gyrus	R	4.92	15	-34	38
			Precuneus	R	4.40	12	-46	56
			Precuneus	R	3.59	12	-55	47
			Middle cingulate cortex	R	3.52	6	-25	35
		77	Supplementary motor area	L	4.82	-3	-4	59

Analyses are conducted using *p* < 0.001 primary and *p*(FWE) = 0.05 secondary cluster extent threshold. *RRS* 10-item Ruminative Response Scale, - negative correlation, *R* right, *L* left, *NA* coordinates are not in AAL

Second, we contrasted stimuli with the same intensity (omitted pain vs. touch) but with different predictive cues. We again found a negative association between rumination score and BOLD signal in the somatosensory cortex (particularly in the postcentral gyrus extending to the midcingulate cortex and precuneus) and SMA (see Table 3 and Fig. 5).

We repeated our analysis adding depressive symptoms as covariate in our model. Results show that it did not alter our previous findings to a great extent using omitted pain versus pain contrast; however, a significant BOLD increase in the inferior parietal lobule (in the supramarginal gyrus) and the inferior frontal gyrus in relation to rumination appeared. In addition, the size of overall activation increased (see Supplementary Table S6). When we analyzed touch versus omitted pain contrast and simultaneously controlled for current depressive symptoms, the previously found association with rumination in cluster with peaks in the postcentral gyrus, precuneus, and midcingulate remained significant, but the size of this cluster decreased (see Supplementary Table S3).

## Discussion

A large amount of evidence suggests that processing of aversive emotional stimuli is altered in trait rumination. This altered emotional processing has been considered as a key mechanism in the maladaptive long-term effects of rumination. Our results extend and specify these previous findings: First, using a classic conditioning paradigm allowed us to disentangle anticipation and perception processes of aversive stimuli and our results show that individual differences in trait rumination modulate anticipatory effects on brain activation to cues predicting aversive (painful) stimuli. Second, we were able to examine brain activation to painful stimuli regardless of predicting cues, and compare it with cue-based processing when congruency between cues and subsequent stimuli was taken into account. Our results support previous findings showing that rumination correlates positively with neural activation to aversive stimuli (painful vs. non-painful stimuli) if we simply analyzed pain perception phase and did not consider cue-stimuli relationship. However, when brain activation to painful versus non-painful stimuli was compared in relation to rumination, congruence/discrepancy between cues and subsequent stimuli had an impact on the relationship. Violated expectations – operationalized either as non-expected pain omission versus expected pain or non-expected non-painful stimuli (omitted pain) versus expected non-painful stimuli – were significantly related to rumination.

### Anticipation of painful stimuli

A recent meta-analysis suggests that anticipation of pain is associated with widespread activation in the brain (Palermo et al., 2015). Contrary to these meta-analytic findings, when the main task effect on anticipation was examined in our study, such a widespread activation was not found; instead only activation and deactivation in occipital areas were revealed (for further discussion, see the Limitations section).

However, when trait rumination was entered in our analysis, widespread brain activation showed a positive correlation with rumination. Thus, people who tend to ruminate respond to pain cues with increased activation in brain areas including the superior temporal gyrus, middle occipital gyrus, inferior parietal lobule, cingulate gyrus, insula, and putamen, which are involved in pain anticipation processes (Palermo et al., 2015), supporting our hypothesis that trait rumination influences anticipatory processes.

We specifically expected that the anterior insula – as a part of the salience network with a clear role in evaluating the biological or psychological/motivational significance of a stimulus and as an area that plays a role in pain processing – would be related to rumination in the anticipation phase. For instance, most of the studies found that the anterior part of the insula plays a role in the anticipation phase of painful (threatening) stimuli (Atlas et al., 2010; Wiech et al., 2010) or in a brief period before the unpleasant stimuli (Ploner, Lee, Wiech, Bingel, & Tracey, 2010). However, there are studies in which recruitment of the middle or posterior part of the insula (Greenberg, Carlson, Rubin, Cha, & Mujica-Parodi, 2015; Schmid et al., 2013) was observed during anticipation of negative outcomes. In our study, insular activity to pain cues in relation to rumination was also located more in the middle subdivisions. Examining the connectivity of the midinsula with other pain-relevant brain regions, Wiech and colleagues (Wiech, Jbabdi, Lin, Andersson, & Tracey, 2014) found that similar to the posterior insula, the midinsula also has structural connections to the primary and secondary somatosensory cortex, and similar to the anterior insula, the midinsula has functional and structural connectivity to the ventrolateral prefrontal cortex (VLPFC). This “hybrid pattern” of connectivity supports the notion that the midinsula plays a role in integrating sensory and cognitive-emotional information (Wiech et al., 2014), and it seems logical that both forms of information are used when processing anticipatory cues.

We also found that rumination correlated with PCC activity – extending to the posterior midcingulate cortex – during the pain anticipation phase. PCC – particularly the ventral PCC (Leech & Sharp, 2014) as a key hub of the default network (Raichle et al., 2001) – has been considered to be involved in increased self-focus and self-referential thinking (see Nejad, Fossati, & Lemogne, 2013), or broadly, in internally driven cognitions (Andrews-Hanna, Smallwood, & Spreng, 2014). Therefore, it is tempting to conclude that detection of cues predicting aversive stimuli increases self-focus and/or internal thoughts to a greater extent among those who tend to ruminate.

It is worth mentioning that when we controlled for current depressive mood the size of the activated clusters decreased, and the activation in PCC had lost its relation to rumination score, suggesting that perception of predicting cues is influenced by current mood state even among healthy adults. We also repeated our analysis with trait anxiety as a control variable, since anxiety has been established to be related to anticipation of threatening stimuli (for a review, see Grupe & Nitschke, 2013). We detected some minor changes: midinsular activation in relation to rumination to pain cues disappeared, but activation to pain cue versus no pain cue in relation to trait rumination in the right VLPFC was detected. The review of Kohn et al. (2014) on neural networks of cognitive emotion regulation posits that VLPFC may signal the need for regulation as a product of the appraisal process. In our study, the duration of cues allowed participants to create conscious expectations and appraise cues in the light of the possible outcome. Our result might suggest that people who tend to ruminate – when trait anxiety is controlled for – appraise threat cues as more demanding, and, thus, the need for regulation of emotions – generated by the threat cue – is more pronounced. Interestingly, Kocsel et al. (2017) found that trait rumination was also associated with increased activation in the VLPFC to rewarding cues, pointing out that information processing of anticipatory cues relating to stimuli evoking heightened arousal – regardless of emotional valence – is exaggerated in trait rumination.

To sum up, our evidence indicates that trait rumination is associated with widespread anticipatory brain activation to pain cue. We propose that this excessive anticipatory response may constitute a potential mechanism through which trait rumination exerts an effect on mental and physical health.

### Perception of pain

Our task activated parts of the pain-processing network including the insula, thalamus, cingulate cortex, postcentral and precentral gyri (rolandic operculum), inferior parietal lobule, and basal ganglia (Apkarian et al., 2005; Tracey & Mantyh, 2007) during the perception of painful stimuli compared to non-painful ones regardless of the contrast (pain vs. touch, pain vs. omitted pain, pain vs. all non-panful stimuli).

#### Relationship between rumination and intensity processing regardless of pain cues

When we simply analyzed pain perception, regardless of predicting cues, we found that trait rumination correlated with activity in the right lateral prefrontal cortex (lPFC) (extending minimally to the medial part of the superior frontal gyrus) and in the parietal lobule (angular gyrus), in the bilateral thalamus to painful stimuli (VAS = 7) versus non-painful stimuli (VAS = 3). The thalamus is one of the key and consistently activated areas in the pain-processing network (Duerden & Albanese, 2013; Jensen et al., 2016), thus our results suggest that rumination is associated with increased neural response to painful stimuli, supporting theories proposing that information processing of aversive (emotional) stimuli is altered or exaggerated in rumination (Koster et al., 2011). It is worth noting that the significant association between rumination and thalamus activation disappeared when current mood was controlled for, suggesting that even variation in healthy mood may influence the perception of painful stimuli.

Theories also suggest that heightened reactivity to aversive stimuli is associated with biased attentional processes (Koster et al., 2011). This idea is indirectly supported by our results showing that painful stimulus activated the right lPFC in relation to rumination. Previous studies found that regardless of the stimulated side of the body, perception of pain was connected to attention-related areas in the right middle frontal gyrus, inferior frontal gyrus, and medial and superior frontal gyri and inferior parietal lobule (Symonds, Gordon, Bixby, & Mande, 2006).

These activations remained significant (and partially extended to VLPFC) in relation to rumination when current mood was controlled for.

#### Congruence/discrepancy

When trait rumination was entered into the analysis we found that congruence between predictive cues and subsequent pain stimulus affected whether rumination correlated with brain activation. When only validly cued stimuli were analyzed, there was no relation between pain perception and trait rumination. Interestingly, when depressive symptoms were controlled for in our additional analysis, activation in lPFC correlated with rumination score positively. It is worth noting that this activation overlapped with activation in the right lateral prefrontal cortex to painful versus non-painful stimuli in relation to rumination.

When discrepancy between cues and stimuli was taken into account, we found that activation in the thalamus, superior/middle frontal gyrus, and PCC (extending to the midcingulate) to omitted pain (unexpected non-painful stimuli) versus pain negatively correlated with rumination. Regarding the thalamus and superior/middle frontal gyrus, our results may simply mean that ruminators respond to painful stimuli in pain processing-related areas and deactivate it to non-painful ones. This notion is supported by another result from our study: there was a significant overlap between the activity of the thalamus and anterolateral prefrontal areas to painful stimuli versus non-painful ones, and the activity of these areas to pain versus omitted pain in relation to rumination.

Decreased activation of the PCC to omitted (but expected) pain was detected as rumination score increased, thus, detection of unexpected violations of an aversive stimulus compared to the delivered stimulus in rumination is associated with a decreased BOLD response in the posterior cingulate cortex. Recently, the PCC has been hypothesized to be a key node in the network responsible for change detection in the environment and subsequent behavioral modification (Pearson, Heilbronner, Barack, Hayden, & Platt, 2011). Thus, it is tempting to conclude that when expectations fail, change (violation) detection occurs to a lesser extent among ruminators, which then prevents subsequent alterations of behavior, possibly contributing to getting stuck in their maladaptive thoughts or to maintaining any maladaptive behaviors. In addition, based on resting state and task-related functional connectivity data, the dorsal part of the PCC is considered to be involved in controlling attentional focus (Leech, Kamourieh, Beckmann, & Sharp, 2011; Leech & Sharp, 2014), thus people with a higher score on the rumination scale may attend to omitted pain to a lesser extent. It is worth noting that rumination-related PCC activity to omitted pain versus pain only partially overlapped with cue-related PCC activity. The latter was more ventral, while omitted pain-related decreased PCC activity was more dorsal, pointing out that different mechanisms may underlie the relationship of rumination with anticipation and with violated expectation of pain.

When depression was controlled for in our analysis, we also found that supramarginal gyrus activity to omitted pain was negatively related to rumination. Of interest, supramarginal gyrus activity to unexpected versus expected sensory events (Bubic, von Cramon, Jacobsen, Schroger, & Schubotz, 2009), including pain (Zeidan et al., 2015) or cognitive events (O'Connor, Han, & Dobbins, 2010) was detected in previous studies. Some evidence suggests that detection of incongruence (or discrepancy) between expectation and perception in supramarginal gyrus is independent of memory-retrieval processes (O'Connor et al., 2010) and is more related to (automatic) reorienting of attention. Interestingly, we found that the angular gyrus is related to omitted pain versus pain across all subjects. This finding is compatible with a recent study showing that the angular gyrus is critically involved in recognition of violated sensory information (Zeidan, Lobanov, Kraft, & Coghill, 2015). Indeed, subjects involved in our analysis could report that one of the cues was followed by painful stimulation but not always. Furthermore, Seghier (2013) argues that the angular gyrus as a cross-modal hub is “an interface between the converging bottom-up multisensory inputs and the top-down predictions” (pp. 52). If there is a difference between the prediction and the perception, a prediction error emerges. There are data suggesting that the angular gyrus is involved in conscious recognition of violated expectations (O'Connor et al., 2010). In light of these results and ideas it is tempting to suggest that the negative correlation between rumination and supramarginal gyrus activity to omitted pain might suggest that detection of a discrepancy between expectations and experience is impaired on a more automatic level.

We also compared brain activity to omitted (but expected) pain (in other words: invalidly cued non-painful stimuli) versus validly cued non-painful stimuli (touch): they had the same intensity (VAS = 3) but different expectations preceded them. Evidence shows that after anticipating a painful stimulus the delivered even non-painful one is experienced as more intense both on a subjective and on a neural level (Sawamoto et al., 2000). This expected pattern was detected in two clusters, namely in the postcentral gyrus (extending to precuneus and midcingulate) and in SMA among those who scored lower on the rumination scale. Activation in the postcentral gyrus overlapped with the primary somatosensory cortex. According to a magnetoencephalography study (Worthen, Hobson, Hall, Aziz, & Furlong, 2011), the primary somatosensory cortex is involved not only in the sensory-discriminating aspect of pain but also in its attentional (affective) aspect as well, suggesting that among non-ruminators, after violated expectation, the non-painful stimuli catch more attention compared to the validly cued non-painful stimuli. It is worth noting that ipsilateral (and not contralateral) primary somatosensory cortex (SI) activation was related to rumination tendency in our study; however, there are data indicating that not just contralateral SI but ipsilateral SI is also activated by somatosensory stimuli (Nihashi et al., 2005). We also found that contralateral SMA – responsible for self-generated movements (Passingham, Bengtsson, & Lau, 2010) – was activated more to omitted pain among those who scored lower on the rumination scale when compared to the same intensity non-painful stimulus. Thus, participants with a higher rumination score gave a lower neural response to invalidly cued non-painful stimuli compared to validly cued non-painful stimuli in areas that are responsible for processing sensory and attentional information and planning motor response. When depressive symptoms were controlled for, activation in the postcentral gyrus – in relation to rumination – remained significant.

### Limitations

Using a long period of rest after stimulation might cause the absence of widespread brain activation to an anticipatory pain cue that has been previously demonstrated in different studies (Palermo et al., 2015). Contrary to these findings, when the main task effect on anticipation was examined in our study, such a widespread activation was not found; instead only activation and deactivation in occipital areas were revealed. Similar minor activation difference to unpleasant electric stimuli cue versus no shock cue was detected by McMenamin and colleagues (McMenamin, Langeslag, Sirbu, Padmala, & Pessoa, 2014) if early temporal factor (5 s after cue onset) was analyzed. Similar to the interpretation of McMenamin et al. (2014), we also speculate that representation of both cues in our study had an important motivational aspect. Pain-predicting cues are inherently important since predicting pain has adaptive functions. Nonetheless, in the context of threat (pain), safety signals could be as salient as pain cues (Christianson et al., 2011). In addition, in most of the pain studies in which anticipation processes were investigated, participants were asked to rate the stimuli (e.g., Atlas et al., 2010; Wiech et al., 2010) or their current state (Drabant et al., 2011) that perhaps made not just the painful stimulus more salient (for a review, see Torta, Legrain, Mouraux, & Valentini, 2017), but the predicting cues as well. It may also happen that the delivered electric stimuli were not fearful enough to elicit anticipatory anxiety across all subjects. However, a robust relationship between self-reported ruminative tendency and anticipatory neural response was detected. In addition, participants did not rate their subjective pain experience, and they were not explicitly asked about their expectation. However, at the end of the scanning session, a post-interview was used to reveal whether they had realized the contingency between cues and stimuli. Only two participants did not notice the contingence, thus their data were excluded from the analysis.

Instead of calculating prediction error with computational models (see Garrison, Erdeniz, & Done, 2013), violated expectation was analyzed as contrasting expected but omitted pain with expected and delivered pain in our study. Using more trials with omission of pain in our experiment would have allowed us to analyse the prediction error component of associative learning. Recent meta-analytical studies (D'Astolfo & Rief, 2017; Garrison et al., 2013) suggest that fronto-striatal circuits are involved in prediction error processing, possibly reflecting the incorporation of new information (i.e., the new predictive value of the cue) into previous expectation. Our results tentatively suggest that trait rumination impairs the use of learning signals generated by a discrepancy between actual and predicted aversive outcomes; however, further support for this notion is required using an analysis in which neural response to prediction error is also calculated. Prospective studies are needed to reveal whether excessive anticipatory processes and/or impaired detection of violated expectations really create a risk for developing psychopathologies.

Furthermore, in our design only non-painful stimuli were expected or unexpected as a function of preceding cues, so we do not know how trait rumination relates to the processing of unexpected painful stimuli. Generally, unexpected pain activates the pain processing network (PPN) more strongly than expected pain (Seidel et al., 2015), and unexpected pain also activates areas outside of the PPN, particularly the angular and supramarginal gyri (Zeidan et al., 2015). It is worth noting that angular gyrus activation to omitted pain versus pain also emerged in our study.

Reinforcement rate in a partial conditioning paradigm may have an effect on neural response to predictive cues. For example, using an explicit uncertain pain cue, i.e. when participants are told that it is uncertain whether a cue might be followed by a painful stimulus (low pain predictability), may lead to higher anticipatory anxiety (Huang, Shang, Dai, & Ma, 2017) and a stronger or different brain response compared to a certain pain cue (Rubio et al., 2015) along with increased pain ratings (Oka et al., 2010). We did not use such an uncertain pain cue in our study; furthermore, our instruction did not contain any explicit information about which cue was followed by which stimulation; our participants had to learn the association between cues and stimuli. However, in light of our results, using uncertain pain cues would add interesting information to the relationship between rumination and anticipation.

### Conclusion

Processing of aversive cues is altered in psychopathologies (Forbes & Goodman, 2014) where rumination constitutes a risk factor (mainly in mood and anxiety disorders, including post-traumatic stress disorder, generalized anxiety disorder, and phobias, but in eating disorders and addiction as well). Thus, based on our results, we hypothesize that expectation-related excessive anticipatory processing under threat of aversive stimuli is one of the processes that may make ruminators more vulnerable to psychopathologies. Our results extend previous evidence on altered (emotional) information processing to the anticipation phase as well.

Moreover, our findings also suggest that detection of violated expectation – as an important change in the environment – is impaired in trait rumination, again constituting a risk factor for psychopathologies. Impaired detection of violated expectations can hinder long-term beneficial effects of psychotherapies in mental disorders. In addition, targeting rumination in cognitive behavioral therapy of chronic pain could also have a beneficial effect, since expectation of pain may contribute to avoidance behavior and lower functioning in patients.

Negative expectations – induced either by verbal suggestions or learning processes as nocebo effects – influence pain perception (Petersen et al., 2014). It is well known that some psychological traits – e.g., fear of pain (Aslaksen & Lyby, 2015) – affect susceptibility to nocebo effects. The results of the present study suggest that inter-individual differences in rumination may also be an important predictor of nocebo effects, and contribute to the exaggerated response to painful stimuli. Therefore, testing the effects of trait rumination on expected pain among chronic pain patients is warranted, since pain expectancy has been demonstrated to predict experienced daily pain (Mun et al., 2017) and disability (Boersma & Linton, 2006) among chronic pain patients.

From the perspective of chronic pain, the rumination component of pain catastrophizing (Sullivan, Bishop, & Pivik, 1995) would also deserve attention. Pain rumination for instance was related to enhanced functional connectivity of medial PFC with thalamus and periaqueductal/periventricular gray in temporomandibular disorders (Kucyi et al., 2014), but we do not know how pain rumination specifically is associated with pain anticipation and perception. Previous studies mainly examined the effect of pain catastrophizing on pain perception (e.g., Mathur et al., 2016), but did not assess how different components – e.g., magnification, rumination and helplessness – contribute to the altered processing of painful stimulus in chronic pain. However, a questionnaire study in fibromyalgia suggests that after the development of a pain disorder pain rumination may have an essential role in shaping the health status of patients (Rodero et al., 2010).

Finally, our results offer new scope for future studies: investigating anticipation processes and related phenomena – for example, when there is a mismatch between expectation and experience – could be a fruitful approach to elucidate mechanisms underlying the adverse effects of rumination on mental and physical health.

## Electronic supplementary material

ESM 1 (DOCX 1.82 mb)
