# Supplementary material for: Anticipation and violated expectation of pain are influenced by trait rumination: An fMRI study
Source: Cogn Affect Behav Neurosci. 2018 Sep 24;19(1):56–72. doi: 10.3758/s13415-018-0644-y (PMC6344394; doi:10.3758/s13415-018-0644-y)
Supplement: Supplementary file 1 — (DOCX 1.82 mb) [file 13415_2018_644_MOESM1_ESM.docx]

**Supplementary materials**

Table S1

*Activation changes during pain anticipation*

| Contrast | Cluster size (voxels) | Region | Side | Peak T-value | MNI coordinates | | |
| --- | --- | --- | --- | --- | --- | --- | --- |
|  |  |  |  |  | x | y | z |
| Pain cue – No pain cue | 258 | Calcarine | R | 7.02 | 9 | -79 | 2 |
|  |  | Lingual Gyrus | R | 6.74 | 6 | -76 | -1 |
|  |  | Lingual Gyrus | L | 6.67 | -6 | -76 | -1 |
|  |  | Calcarine | L | 5.28 | -9 | -88 | 5 |
|  |  | Calcarine | L | 4.99 | -6 | -91 | 11 |
| No pain cue – Pain cue | 137 | Middle Occipital Gyrus | L | 7.21 | -30 | -88 | 5 |
|  |  | Inferior Occipital Gyrus | L | 4.53 | -36 | -76 | -7 |
|  | 157 | Middle Occipital Gyrus | R | 7.01 | 30 | -82 | 2 |
|  |  | Lingual Gyrus | R | 5.09 | 24 | -82 | -10 |

*Note.* Analyses are conducted using *p* < 0.001 primary and *p*(FWE) = 0.05 secondary cluster extent threshold. R: right; L: left


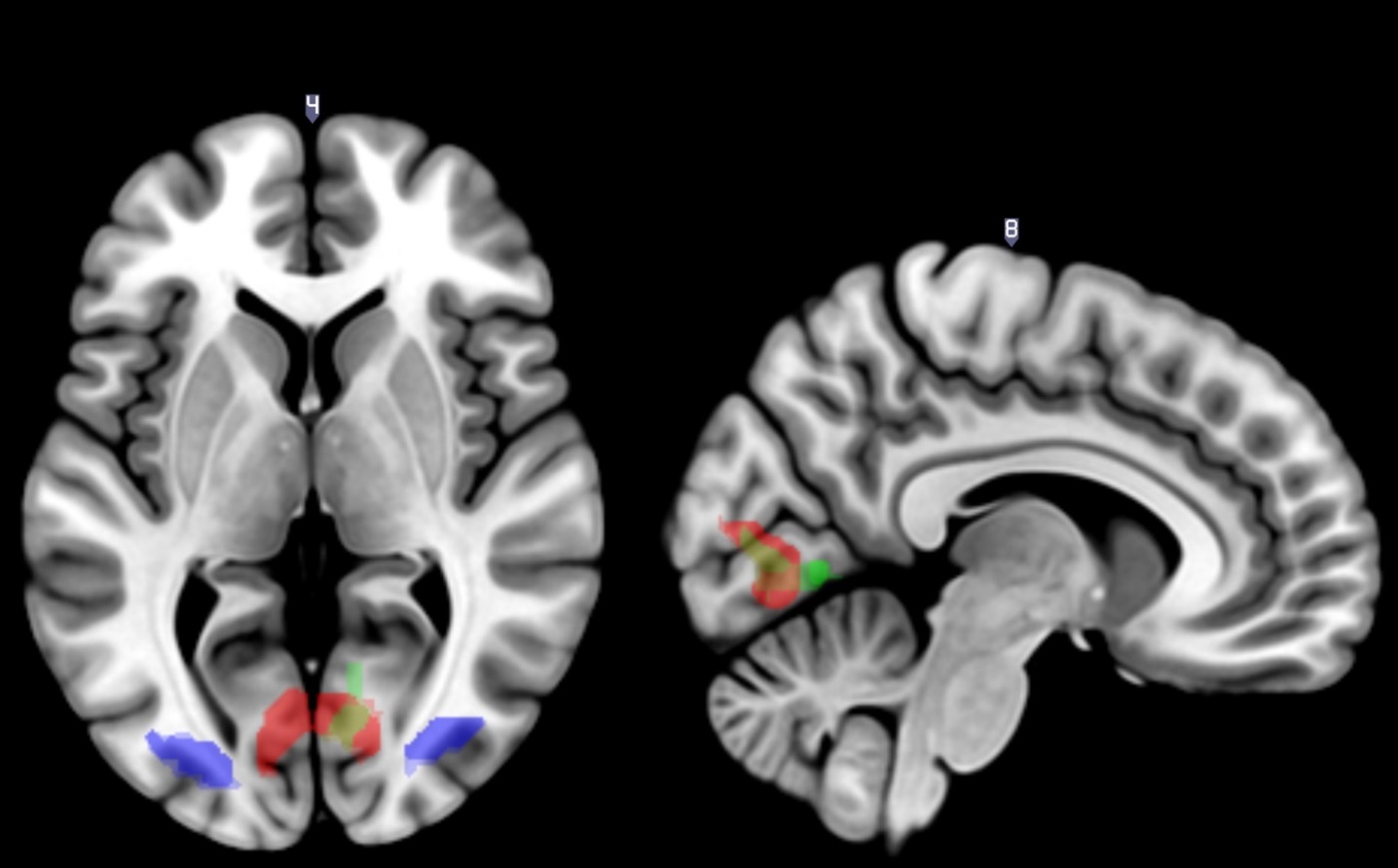


*Figure S1*. Occipital activation changes during pain anticipation at *p* < 0.001 level. Red: increased activation, blue: decreased activation. Deactivation to omitted pain vs. touch (green) partially overlaps with increased occipital activation to pain cue (x = 8, y = 4).


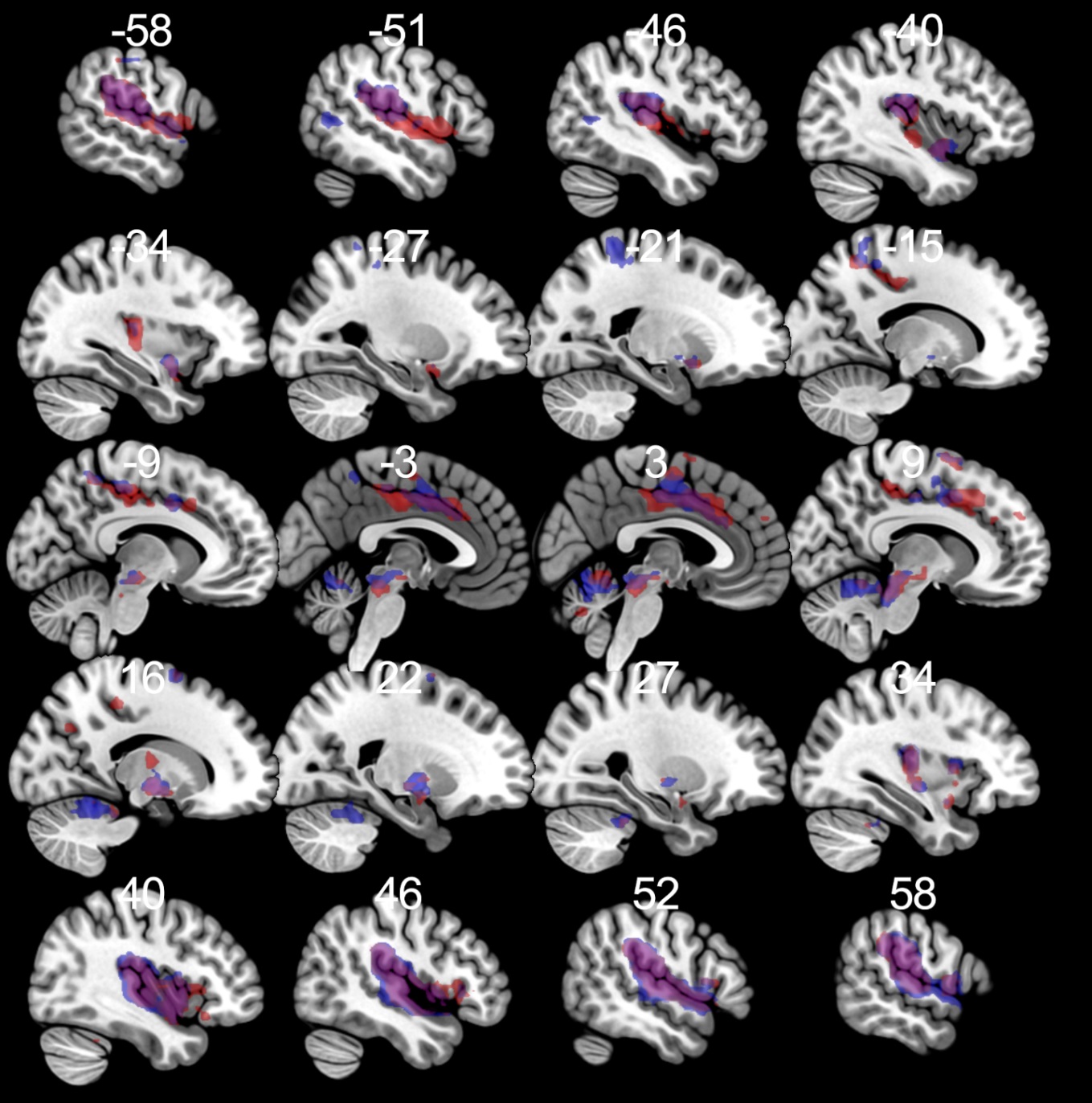


*Figure S2*. Painful vs. non-painful (blue) and painful vs. validly cued non-painful (touch) stimuli (red) contrasts and the overlapping activation (purple) at p < 0.001 threshold for illustrative purposes with a minimum cluster size of 10 voxels.

Table S2

*Activation changes to painful versus non-pain (touch) stimulation that is cue congruent (A) and to painful and non-painful stimulation that disregard the preceding cues (B)*

| 1. Contrast | Cluster size (voxels) | Region | | Side | | Peak T-value | MNI coordinates | | | | | |  |
| --- | --- | --- | --- | --- | --- | --- | --- | --- | --- | --- | --- | --- | --- |
|  |  |  |  |  |  |  | x | | y | | z | |  |
| Pain - Touch | 1590 | Insula | | R | | 8.75 | 39 | | -19 | | 14 | |  |
|  |  | Insula | | R | | 7.61 | 45 | | 2 | | -7 | |  |
|  |  | Supramarginal Gyrus | | R | | 7.44 | 60 | | -34 | | 29 | |  |
|  |  | Insula | | R | | 6.94 | 39 | | -19 | | 2 | |  |
|  |  | Insula | | R | | 6.78 | 42 | | 5 | | 2 | |  |
|  |  | Insula | | R | | 6.78 | 45 | | -10 | | -1 | |  |
|  |  | Midbrain | | R | | 6.44 | 6 | | -31 | | -16 | |  |
|  |  | NA | | R | | 6.05 | 39 | | 5 | | -16 | |  |
|  |  | NA | | R | | 6.05 | 15 | | -13 | | -7 | |  |
|  |  | NA | | R | | 5.78 | 48 | | -28 | | 29 | |  |
|  |  | Rolandic Operculum | | R | | 5.36 | 51 | | -28 | | 20 | |  |
|  |  | Midbrain | | L | | 4.96 | -3 | | -28 | | -16 | |  |
|  |  | Rolandic Operculum | | R | | 4.84 | 57 | | 8 | | 8 | |  |
|  |  | Thalamus | | R | | 4.75 | 15 | | -10 | | 11 | |  |
|  |  | Inferior Frontal Gyrus pars Triangularis | | R | | 4.39 | 42 | | 23 | | 5 | |  |
|  |  | Cerebellum | | R | | 4.34 | 12 | | -34 | | -25 | |  |
|  | 918 | Rolandic Operculum | | L | | 7.84 | -39 | | -19 | | 17 | |  |
|  |  | Insula | | L | | 7.80 | -39 | | -19 | | 11 | |  |
|  |  | Supramarginal Gyrus | | L | | 7.23 | -63 | | -25 | | 20 | |  |
|  |  | Superior Temporal Gyrus | | L | | 7.17 | -57 | | -31 | | 17 | |  |
|  |  | Rolandic Operculum | | L | | 6.06 | -51 | | -7 | | 2 | |  |
|  |  | Rolandic Operculum | | L | | 5.31 | -39 | | -31 | | 17 | |  |
|  |  | Insula | | L | | 4.49 | -39 | | -19 | | -4 | |  |
|  |  | NA | | L | | 4.41 | -36 | | -22 | | -1 | |  |
|  |  | Middle Temporal Gyrus | | L | | 4.12 | -63 | | -28 | | 41 | |  |
|  |  | Inferior Frontal Gyrus pars Opercularis | | L | | 3.95 | -57 | | 11 | | 5 | |  |
|  |  | Inferior Parietal Gyrus | | L | | 3.80 | -60 | | -25 | | 44 | |  |
|  |  | Inferior Parietal Gyrus | | L | | 3.77 | -57 | | -34 | | 47 | |  |
|  | 599 | Middle Cingulate Cortex | | L | | 6.18 | -9 | | -22 | | 47 | |  |
|  |  | Middle Cingulate Cortex | | R | | 6.13 | 3 | | 5 | | 41 | |  |
|  |  | Paracentral Lobule | | R | | 5.32 | 12 | | -34 | | 50 | |  |
|  |  | Middle Cingulate Cortex | | - | | 5.16 | 0 | | -13 | | 41 | |  |
|  |  | Middle Cingulate Cortex | | R | | 5.05 | 3 | | 20 | | 32 | |  |
|  |  | Middle Cingulate Cortex | | L | | 5.04 | -3 | | 17 | | 35 | |  |
|  |  | Precuneus | | L | | 4.77 | -12 | | -55 | | 53 | |  |
|  |  | Precuneus | | L | | 4.03 | -6 | | -49 | | 56 | |  |
|  |  | Precuneus | | L | | 3.75 | -9 | | -46 | | 53 | |  |
|  |  | Precuneus | | L | | 3.72 | -15 | | -46 | | 53 | |  |
|  | 100 | Insula | | L | | 5.51 | -39 | | -1 | | -13 | |  |
|  |  | NA | | L | | 5.34 | -30 | | 5 | | -19 | |  |
|  |  | NA | | L | | 4.53 | -21 | | 8 | | -13 | |  |
|  |  | Insula | | L | | 3.50 | -42 | | 14 | | -10 | |  |
| 1. Contrast | Cluster size (voxels) | Region | Side | | Peak T-value | | | MNI coordinates  x y z | | | | | |
| Painful – Non-painful stimuli (touch + | 2045 | Insula | R | | 9.11 | | | 45 | | 2 | | -7 | |
|  |  | Supramarginal Gyrus | R | | 8.22 | | | 60 | | -25 | | 23 | |
| omitted pain) |  | Insula | R | | 8.18 | | | 39 | | -19 | | 14 | |
|  |  | Supramarginal Gyrus | R | | 7.91 | | | 63 | | -28 | | 26 | |
|  |  | NA | R | | 7.81 | | | 48 | | -28 | | 29 | |
|  |  | Insula | R | | 7.40 | | | 39 | | -19 | | -1 | |
|  |  | Heschl Gyrus | R | | 7.39 | | | 51 | | -16 | | 8 | |
|  |  | Insula | R | | 6.76 | | | 45 | | -10 | | -1 | |
|  |  | NA | R | | 6.55 | | | 18 | | -7 | | -4 | |
|  |  | Cerebellum | R | | 6.14 | | | 27 | | -37 | | -28 | |
|  |  | NA | R | | 6.07 | | | 6 | | -28 | | -13 | |
|  |  | Cerebellum | R | | 5.56 | | | 12 | | -55 | | -16 | |
|  |  | Cerebellum | R | | 5.40 | | | 15 | | -49 | | -19 | |
|  |  | Rolandic Operculum | R | | 5.37 | | | 57 | | 5 | | 2 | |
|  |  | Insula | R | | 4.97 | | | 36 | | 11 | | 8 | |
|  |  | Cerebellum | R | | 4.90 | | | 0 | | -61 | | -16 | |
|  | 657 | Superior Temporal Gyrus | L | | 7.39 | | | -51 | | -34 | | 20 | |
|  |  | Supramarginal Gyrus | L | | 7.21 | | | -54 | | -28 | | 20 | |
|  |  | Superior Temporal Gyrus | L | | 7.14 | | | -57 | | -31 | | 17 | |
|  |  | Rolandic Operculum | L | | 6.97 | | | -39 | | -22 | | 20 | |
|  |  | Supramarginal Gyrus | L | | 6.81 | | | -63 | | -25 | | 20 | |
|  |  | Superior Temporal Gyrus | L | | 6.39 | | | -57 | | -25 | | 11 | |
|  |  | Rolandic Operculum | L | | 4.91 | | | -57 | | 2 | | 2 | |
|  |  | Superior Temporal Gyrus | L | | 4.53 | | | -57 | | -4 | | 2 | |
|  |  | Supramarginal Gyrus | L | | 4.05 | | | -63 | | -28 | | 41 | |
|  |  | Postcentral Gyrus | L | | 3.73 | | | -57 | | -19 | | 47 | |
|  |  | Superior Temporal Pole | L | | 3.60 | | | -57 | | 11 | | -7 | |
|  | 497 | Middle Cingulate Cortex | - | | 6.78 | | | 0 | | 2 | | 41 | |
|  |  | Middle Cingulate Cortex | R | | 5.02 | | | 3 | | -10 | | 47 | |
|  |  | Superior Parietal Gyrus | L | | 4.86 | | | -21 | | -46 | | 68 | |
|  |  | Paracentral Lobule | L | | 4.56 | | | -6 | | -28 | | 50 | |
|  |  | Precuneus | L | | 4.48 | | | -12 | | -55 | | 56 | |
|  |  | Precuneus | L | | 4.23 | | | -6 | | -49 | | 56 | |
|  |  | NA | L | | 4.21 | | | -21 | | -40 | | 56 | |
|  |  | Supplementary Motor Area | L | | 4.15 | | | 9 | | -4 | | 53 | |
|  |  | NA | L | | 4.12 | | | -24 | | -34 | | 53 | |
|  |  | Middle Cingulate Cortex | L | | 4.04 | | | -15 | | -40 | | 53 | |
|  |  | Precuneus | R | | 3.94 | | | -15 | | -52 | | 65 | |
|  | 103 | Insula | L | | 6.65 | | | -39 | | -1 | | -13 | |
|  |  | Putamen | L | | 4.53 | | | -21 | | 8 | | -10 | |
|  |  | Pallidum | L | | 3.94 | | | -18 | | -7 | | -7 | |

Note. Analyses are conducted using *p* < 0.001 primary and *p*(FWE) = 0.05 secondary cluster extent threshold. R: right; L: left; NA: coordinates are not in AAL.


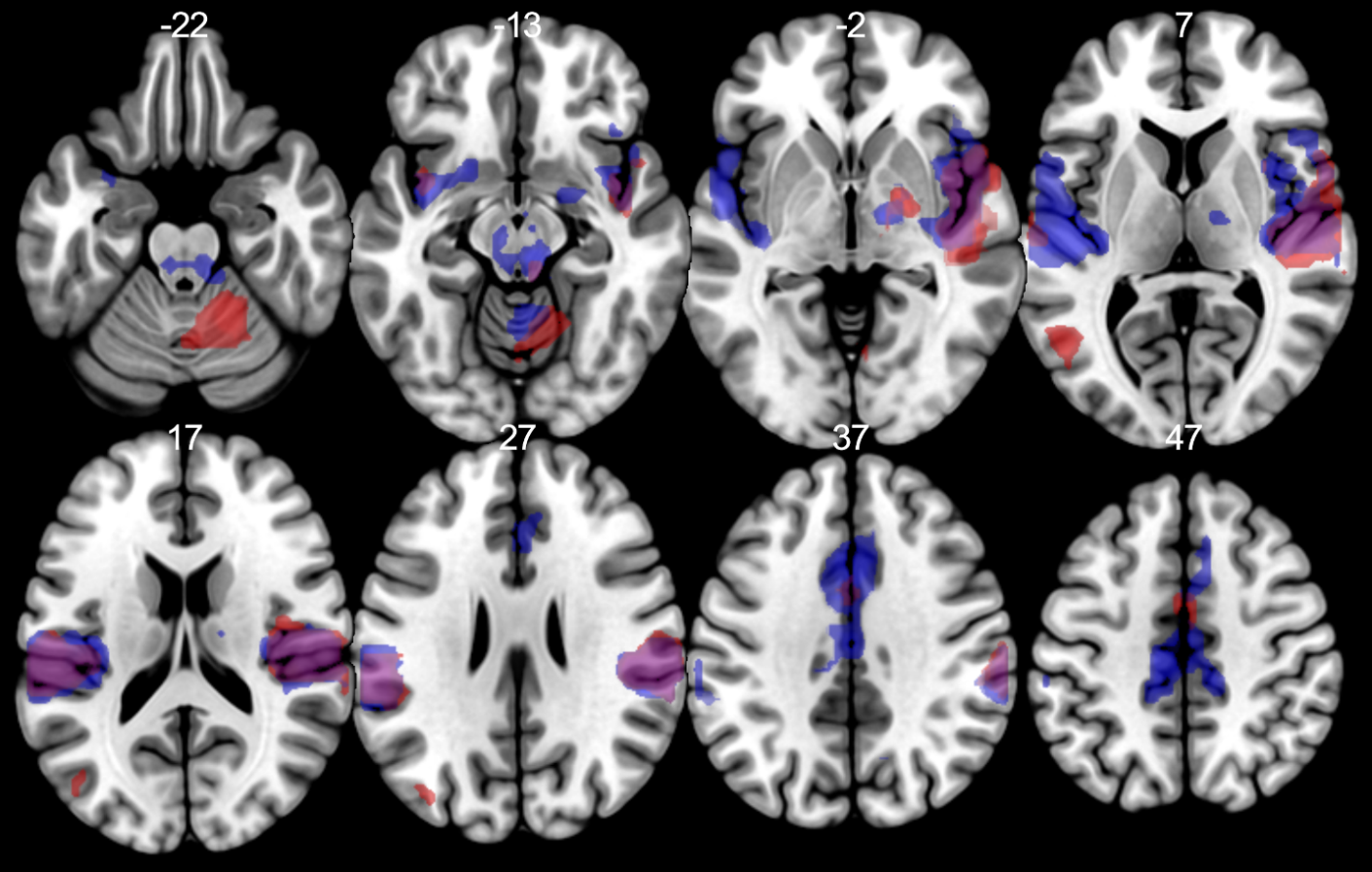


*Figure S3*. Pain vs. omitted pain (red) and pain vs. touch (blue) contrasts and the overlapping activation (purple) at *p* < 0.001 threshold for illustrative purposes with a minimum cluster size of 10 voxels.

Table S3

*Activation changes to omitted pain vs. pain*

| Contrast | Cluster size (voxels) | Region | Side | Peak T-value | MNI coordinates | | |
| --- | --- | --- | --- | --- | --- | --- | --- |
|  |  |  |  |  | x | y | z |
| Omitted pain - Pain | 75 | Angular Gyrus | R | 8.09 | 48 | -61 | 38 |
|  |  | Angular Gyrus | R | 7.55 | 45 | -61 | 44 |
|  |  | Angular Gyrus | R | 7.24 | 42 | -58 | 32 |
| Pain - Omitted pain | 1228 | Supramarginal Gyrus | R | 6.86 | 48 | -28 | 26 |
|  |  | Supramarginal Gyrus | R | 6.46 | 60 | -25 | 23 |
|  |  | Insula | R | 6.27 | 45 | 2 | -7 |
|  |  | Heschl Gyrus | R | 6.07 | 45 | -22 | 11 |
|  |  | Superior Temporal Gyrus | R | 5.86 | 42 | -19 | -1 |
|  |  | Superior Temporal Gyrus | R | 5.76 | 48 | -13 | -1 |
|  |  | Rolandic Operculum | R | 5.62 | 39 | -13 | 17 |
|  |  | Supramarginal Gyrus | R | 4.98 | 63 | -25 | 35 |
|  |  | Rolandic Operculum | R | 4.67 | 57 | 5 | 2 |
|  |  | NA | R | 4.39 | 51 | 11 | -7 |
|  |  | Insula | R | 6.64 | 42 | -10 | -10 |
|  |  | NA | R | 5.59 | 39 | -28 | -1 |
|  |  | Superior Temporal Gyrus | R | 5.50 | 66 | -34 | 11 |
|  | 380 | Supramarginal Gyrus | L | 5.38 | -54 | -28 | 23 |
|  |  | Postcentral Gyrus | L | 4.98 | -51 | -19 | 20 |
|  |  | Superior Temporal Gyrus | L | 3.71 | -60 | -22 | 11 |
|  |  | Postcentral Gyrus | L | 3.60 | -54 | -16 | 17 |
|  |  | Rolandic Operculum | L | 6.46 | -39 | -25 | 17 |
|  |  | Rolandic Operculum | L | 4.56 | -60 | -1 | 5 |
|  |  | Rolandic Operculum | L | 4.49 | -57 | 2 | 2 |
|  | 250 | Cerebellum | R | 3.82 | 18 | -49 | -25 |
|  |  | Cerebellum | R | 3.72 | 3 | -61 | -25 |
|  |  | Cerebellum | - | 8.09 | 0 | -64 | -19 |
|  |  | Cerebellum | R | 7.55 | 3 | -70 | -7 |
|  |  | Lingual Gyrus | R | 7.24 | 6 | -67 | -4 |
| Omitted pain - Touch |  | No significant activation |  |  |  |  |  |
| Touch - Omitted | 74 | Calcarine | R | 4.47 | 6 | -79 | 5 |
| Pain |  | Lingual Gyrus | R | 4.39 | 9 | -64 | -1 |
|  |  | Lingual Gyrus | R | 4.27 | 9 | -73 | -1 |

Note. Analyses are conducted using *p* < 0.001 primary and *p*(FWE) = 0.05 secondary cluster extent threshold. R: right; L: left. NA: coordinates are not in AAL.

Table S4

*Anticipation of pain in relation to rumination controlled for current depressive symptoms*

| Contrast | RRS | Cluster size (voxels) | Region | Side | Peak T- value | MNI Coordinates | | |
| --- | --- | --- | --- | --- | --- | --- | --- | --- |
|  |  |  |  |  |  | x y z | | |
| Pain cue vs. | + | 66 | Inferior Temporal Gyrus | L | 5.95 | -42 | -61 | -7 |
| No Pain cue |  |  | Inferior Temporal Gyrus | L | 4.28 | -48 | -61 | -13 |
|  |  | 244 | Paracentral Lobule | R | 5.41 | 12 | -31 | 56 |
|  |  |  | NA | L | 4.86 | -18 | -22 | 47 |
|  |  |  | Postcentral Gyrus | L | 4.79 | -27 | -34 | 47 |
|  |  |  | Midcingulate Cortex | L | 4.67 | -9 | -22 | 47 |
|  |  |  | NA | L | 4.47 | -27 | -7 | 44 |
|  |  |  | Postcentral Gyrus | L | 4.44 | -24 | -37 | 50 |
|  |  |  | SMA | L | 4.16 | 0 | -16 | 53 |
|  |  |  | SMA | L | 4.07 | 0 | -4 | 53 |
|  |  |  | SMA | R | 3.83 | 3 | -22 | 50 |
|  |  |  | SMA | R | 3.70 | 6 | -22 | 59 |
|  |  | 116 | Insula | R | 5.22 | 45 | 5 | -1 |
|  |  |  | Rolandic Operculum | R | 4.61 | 54 | -10 | 14 |
|  |  |  | NA | R | 4.40 | 36 | -7 | -4 |
|  |  |  | Rolandic Operculum | R | 4.14 | 48 | 5 | 11 |
|  |  |  | Putamen | R | 4.12 | 33 | -10 | -7 |
|  |  | 186 | NA | R | 5.19 | 42 | -40 | 5 |
|  |  |  | Superior Temporal Gyrus | R | 4.98 | 57 | -37 | 8 |
|  |  |  | Middle Temporal Gyrus | R | 4.64 | 45 | -61 | 14 |
|  |  |  | NA | R | 4.59 | 42 | -49 | -1 |
|  |  |  | Fusiform Gyrus | R | 3.98 | 42 | -49 | 13 |
|  |  | 71 | Inferior Parietal Lobule | R | 4.91 | 39 | -49 | 47 |
|  |  |  | Supramarginal Gyrus | R | 4.75 | 51 | -40 | 41 |
|  |  | 177 | Superior Occipital Gyrus | L | 4.52 | -21 | -70 | 29 |
|  |  |  | Superior Parietal Lobule | L | 4.47 | -15 | -70 | 47 |
|  |  |  | Precuneus | L | 4.38 | -15 | -58 | 50 |
|  |  |  | Middle Occipital Lobule | L | 4.18 | -36 | -67 | 23 |
|  |  |  | Middle Occipital Lobule | L | 4.15 | -36 | -70 | 17 |
|  |  |  | Cuneus | L | 4.04 | -24 | -73 | 14 |
|  |  |  | Inferior Parietal Lobule | L | 3.90 | -24 | -67 | 41 |
|  |  |  | Precuneus | L | 3.83 | -18 | -58 | 35 |
|  |  |  | NA | L | 3.71 | -33 | -55 | 26 |
|  |  |  | Middle Occipital Gyrus | L | 3.69 | -30 | -73 | 38 |
|  |  |  | Inferior Parietal Lobule | L | 3.66 | -30 | -52 | 38 |

Note. Analyses are conducted using p < 0.001 primary and p(FWE) = 0.05 secondary cluster extent threshold. RRS: 10-item Ruminative Response Scale; +: positive correlation. R: right; L: left. SMA: Supplementary Motor Area. NA: coordinates are not in AAL.

Table S5

*Anticipation of pain in relation to rumination controlled for trait anxiety*

| Contrast | RRS | Cluster size  (voxels) | Region | Side | Peak T- value | MNI Coordinates | | |
| --- | --- | --- | --- | --- | --- | --- | --- | --- |
|  |  |  |  |  |  | x | y | z |
| Pain cue vs. | + | 69 | Inferior Temporal Gyrus | L | 6.01 | -42 | -61 | -7 |
| No Pain cue |  |  | Inferior Temporal Gyrus | L | 4.31 | -48 | -61 | -13 |
|  |  | 296 | Superior Temporal Gyrus | R | 5.51 | 30 | -67 | 17 |
|  |  |  | Superior Temporal Gyrus | R | 5.42 | 42 | -49 | -1 |
|  |  |  | Superior Temporal Gyrus | R | 5.40 | 42 | -40 | 5 |
|  |  | 106 | Inferior Parietal Lobule | R | 5.26 | 39 | -49 | 44 |
|  |  |  | Supramarginal Gyrus | R | 4.54 | 51 | -40 | 41 |
|  |  |  | Superior Parietal Lobule | R | 3.94 | 33 | -64 | 50 |
|  |  | 168 | Paracentral Lobule | R | 5.21 | 12 | -31 | 56 |
|  |  |  | Paracentral Lobule | L | 4.87 | -18 | -22 | 47 |
|  |  |  | Postcentral Gyrus | L | 4.70 | -27 | -34 | 47 |
|  |  | 338 | Precuneus | L | 5.00 | -12 | -70 | 47 |
|  |  |  | Superior Occipital Lobule | L | 4.51 | -21 | -70 | 29 |
|  |  |  | Precuneus | L | 4.51 | -15 | -58 | 50 |
|  |  | 111 | Inferior Frontal Gyrus, pars triangularis | R | 4.77 | 48 | 20 | 23 |
|  |  |  | Inferior Frontal Gyrs,  Pars opercularis | R | 4.50 | 42 | 8 | 26 |
|  |  |  | Inferior Frontal Gyrs,  Pars opercularis | R | 4.48 | 30 | 14 | 38 |
|  |  | 90 | Posterior Cingulate | L | 4.57 | -3 | -49 | 20 |
|  |  | 107 | Precuneus | R | 4.44 | 15 | -55 | 44 |
|  |  |  | Superior Parietal Lobule | R | 3.71 | 18 | -52 | 59 |
|  |  |  | Midcingulum | R | 3.50 | 15 | -43 | 35 |

Note. Analyses are conducted using p < 0.001 primary and p(FWE) = 0.05 secondary cluster extent threshold. RRS: 10-item Ruminative Response Scale; +: positive correlation. R: right; L: left.

Table S6

*Perception of pain in relation to rumination controlled for current depressive symptoms*

| Contrast | RRS | Cluster size  (voxels) | Region | Side | Peak T- value | MNI Coordinates | | |
| --- | --- | --- | --- | --- | --- | --- | --- | --- |
|  |  |  |  |  |  | x | y | z |
| Pain vs. touch | + | 68 | Middle Frontal Gyrus | R | 4.85 | 36 | 56 | 5 |
|  |  |  | Superior Frontal Gyrus | R | 4.37 | 21 | 59 | 8 |
|  |  |  | Superior Frontal Gyrus | R | 4.24 | 27 | 50 | 17 |
|  |  |  | Middle Frontal Gyrus | R | 3.65 | 45 | 50 | 2 |
| Painful vs. | + | 203 | Superior Frontal Gyrus | R | 5.50 | 21 | 59 | 8 |
| Non-painful |  |  | Middle Frontal Gyrus | R | 5.41 | 39 | 53 | 2 |
|  |  |  | Inferior Frontal Gyrus | R | 5.33 | 42 | 47 | 2 |
|  |  |  | Superior Frontal Gyrus | R | 4.96 | 24 | 62 | 2 |
|  |  |  | Superior Frontal Gyrus | R | 4.74 | 30 | 53 | 14 |
|  |  |  | Middle Frontal Gyrus | R | 4.62 | 27 | 50 | 17 |
|  |  |  | Middle Frontal Gyrus | R | 4.51 | 36 | 50 | 20 |
|  |  |  | Superior Frontal Gyrus | R | 3.84 | 21 | 53 | -1 |
|  |  |  | Middle Frontal Gyrus | R | 3.66 | 33 | 41 | 17 |
|  |  | 61 | Inferior Frontal Gyrus,  pars opercularis | R | 5.01 | 42 | 11 | 14 |
|  |  |  | NA | R | 4.04 | 30 | 23 | 14 |
|  |  | 136 | Inferior Parietal Lobule | R | 4.96 | 45 | -55 | 50 |
|  |  |  | Inferior Parietal Lobule | R | 4.91 | 45 | -49 | 47 |
|  |  |  | Angular Gyrus | R | 4.81 | 42 | -61 | 50 |
|  |  |  | Inferior Parietal Lobule | R | 4.65 | 36 | -55 | 47 |
|  |  |  | Supramarginal Gyrus | R | 4.54 | 54 | -43 | 44 |
|  |  |  | Angular Gyrus | R | 4.47 | 36 | -64 | 47 |
|  |  | 73 | Middle Frontal Gyrus | R | 4.83 | 36 | -1 | 62 |
|  |  |  | Superior Frontal Gyrus | R | 4.48 | 33 | -4 | 65 |
|  |  |  | Precentral Gyrus | R | 4.37 | 48 | 2 | 44 |
|  |  |  | Frontal Superior Gyrus | R | 4.30 | 24 | 5 | 62 |
|  |  |  | Precentral Gyrus | R | 4.04 | 45 | 2 | 50 |
|  |  |  | Middle Frontal Gyrus | R | 3.86 | 42 | 2 | 59 |
|  |  |  | Middle Frontal Gyrus | R | 3.85 | 30 | 8 | 53 |
|  |  |  | Superior Frontal Gyrus | R | 3.75 | 24 | 11 | 53 |
| Omitted Pain | - | 269 | Posterior Cingulate | R | 5.74 | 3 | -37 | 23 |
| vs. Pain |  |  | Midcingulate | R | 4.64 | 9 | -28 | 41 |
|  |  |  | Midcingulate |  | 4.47 | 0 | -25 | 32 |
|  |  |  | Midcingulate | L | 4.17 | -12 | -31 | 38 |
|  |  |  | Midcingulate | L | 3.98 | -15 | -43 | 35 |
|  |  |  | Midcingulate | R | 3.66 | 18 | -37 | 41 |
|  |  | 153 | Thalamus | L | 5.73 | -3 | -16 | 17 |
|  |  |  | Thalamus | L | 4.45 | -12 | -25 | 17 |
|  |  |  | Caudate | L | 4.17 | -6 | 5 | 14 |
|  |  |  | Thalamus | L | 4.08 | -3 | -7 | 11 |
|  |  |  | NA | R | 4.00 | 18 | -34 | 14 |
|  |  |  | Caudate | L | 3.90 | -15 | -7 | 20 |
|  |  |  | NA | L | 3.82 | -15 | -37 | 14 |
|  |  | 181 | Middle Frontal Gyrus | R | 5.48 | 42 | 47 | 2 |
|  |  |  | Superior Frontal Gyrus | R | 5.10 | 21 | 59 | 8 |
|  |  |  | Superior Frontal Gyrus | R | 5.06 | 24 | 62 | 2 |
|  |  |  | Superior Frontal Gyrus | R | 4.69 | 27 | 59 | 11 |
|  |  |  | Superior Frontal Gyrus | R | 4.64 | 21 | 53 | -1 |
|  |  |  | Middle Frontal Gyrus | R | 4.20 | 36 | 50 | 20 |
|  |  |  | Middle Frontal Gyrus | R | 4.05 | 30 | 50 | 17 |
|  |  |  | Middle Frontal Gyrus | R | 4.02 | 36 | 50 | 11 |
|  |  |  | Middle Frontal Gyrus | R | 3.74 | 33 | 41 | 17 |
|  |  | 128 | Inferior Parietal Lobule | R | 5.18 | 42 | -55 | 47 |
|  |  |  | Inferior Parietal Lobule | R | 4.93 | 36 | -55 | 44 |
|  |  |  | Supramarginal Gyrus | R | 3.96 | 57 | -43 | 38 |
|  |  |  | Supramarginal Gyrus | R | 3.75 | 48 | -43 | 35 |
|  |  | 67 | Middle Frontal Gyrus | R | 5.14 | 42 | 17 | 35 |
|  |  |  | Middle Frontal Gyrus | R | 4.22 | 45 | 20 | 47 |
|  |  |  | Middle Frontal Gyrus | R | 3.93 | 45 | 32 | 35 |
|  |  |  | Middle Frontal Gyrus | R | 3.69 | 33 | 8 | 38 |
|  |  |  | Middle Frontal Gyrus | R | 3.60 | 36 | 11 | 41 |
|  |  | 66 | NA | R | 4.87 | 39 | 11 | 20 |
|  |  |  | Inferior Frontal Gyrus, pars opercularis | R | 4.52 | 48 | 14 | 17 |
|  |  |  | NA | R | 4.46 | 45 | 8 | 17 |
|  |  |  | NA | R | 4.31 | 36 | 8 | 23 |
|  |  |  | Inferior Frontal Gyrus, parts triangularis | R | 3.62 | 57 | 20 | 23 |
| Touch vs. | - | 78 | Postcentral Gyrus | R | 5.06 | 27 | -43 | 50 |
| Omitted pain |  |  | Midcingulate | R | 4.72 | 15 | -34 | 38 |
|  |  |  | Midcingulate | R | 4.60 | 15 | -34 | 56 |
|  |  |  | Precuneus | R | 4.42 | 12 | -46 | 56 |

Note. Analyses are conducted using p < 0.001 primary and p(FWE) = 0.05 secondary cluster extent threshold. RRS: 10-item Ruminative Response Scale; +: positive correlation; -: negative correlation; R: right; L: left. NA: coordinates are not in AAL.
